# The Effect of Low-Intensity Daily Walking Activity on Cognitive and Brain Function in Older Adults

**DOI:** 10.1093/geroni/igab046.2887

**Published:** 2021-12-17

**Authors:** Dana Eldreth, Vijay Varma, Yi-Fang Chuang, Michelle Carlson

**Affiliations:** 1 Johns Hopkins Bloomberg School of Public Health, Baltimore, Maryland, United States; 2 National Institute on Aging, Baltimore, Maryland, United States; 3 National Yang Ming Chiao Tung University, Taipei, Taipei, Taiwan (Republic of China); 4 Johns Hopkins University, Baltimore, Maryland, United States

## Abstract

Physical activity is an effective intervention to prevent or delay cognitive decline and dementia in older adults; however, many have difficulty achieving recommended moderate- to vigorous-intensity guidelines. This study examined the impact of low-intensity daily walking activity on executive cognitive and brain function in 66 older adults (mean age=67.26 ; SD=6.04). Daily walking activity was measured using a step activity monitor and brain function was assessed using functional magnetic resonance imaging during the Flanker task. Analyses included whole and region of interest (ROI) in the right middle frontal gyrus (RMFG), occipital cortex (OCC) and anterior cingulate (ACC). Partial correlations were performed between step activity, behavioral performance, and ROI activation, adjusting for age and education. Most of the step activity was in the low-intensity range. No associations were observed between step activity and task performance (p>.05). Task-related activation occurred in the RMFG, lateral OCC and paracingulate (p<.01). Increased activation in the RMFG was associated with greater amount r(62)=.390, p=.001, duration r(62)=.309, p=.013 and frequency r(62)=.327, p=.007 of step activity. Stratification by sex revealed a positive association between amount of step activity and RMFG activation in women r(44)= .360, p=.014, but not men. Whole brain correlation revealed that amount of step activity was positively associated with precuneus activation (p<.01), an area impacted early in Alzheimer’s disease. These results support the benefits of low intensity daily walking activity on prefrontal function in older adults and suggest the importance of designing attainable and sustainable physical activity interventions to promote brain health in older adults.

